# Internet Use, Social Networks, and Loneliness Among the Older Population in China

**DOI:** 10.3389/fpsyg.2022.895141

**Published:** 2022-05-12

**Authors:** Dan Tang, Yongai Jin, Kun Zhang, Dahua Wang

**Affiliations:** ^1^Center for Population and Development Studies, Renmin University of China, Beijing, China; ^2^Institute of Gerontology, Renmin University of China, Beijing, China; ^3^National Institute of Education Sciences, Beijing, China; ^4^Faculty of Psychology, Beijing Normal University, Beijing, China

**Keywords:** Internet use, social networks, loneliness, older adults, China

## Abstract

While the rate of Internet use among the older population in China is rapidly increasing, the outcomes associated with Internet use remain largely unexplored. Currently, there are contradictory findings indicating that Internet use is sometimes positively and sometimes negatively associated with older adults’ subjective well-being. Therefore, we examined the associations between different types of Internet use, social networks, and loneliness among Chinese older adults using data from the Chinese Longitudinal Ageing Society Survey (*N* = 1863). Internet use was classified as interpersonal communication and information acquisition, and social networks were divided into family and friendship ties. The results showed that both interpersonal communication and information acquisition were associated with lower loneliness. Interpersonal communication can increase social networks, and family ties have a mediating effect on the association between Internet use for interpersonal communication and loneliness. Although information acquisition can directly decrease loneliness in older adults, it can also damage existing social networks and further increase loneliness. Family ties act as a suppressor in the association between Internet use for information acquisition and loneliness. Our study further discusses important implications for improving the subjective well-being of older adults in the digital era, based on the empirical findings.

## Introduction

As of January 2021, 59.5% of the global population were active Internet users^1^ and the corresponding rate for China was 71.6%,^2^ placing it far ahead of many developing countries in the world. In recent years, Internet use has also exploded in popularity among the older population in China. The proportion of the older population aged 60 or above who use the Internet increased from 4.8% in 2010 to 42% in December 2020.^3^ The rapidity with which the Internet has become a mainstream medium in modern life has tremendously changed every aspect of society. One of the most prominent social consequences of Internet use is its effect on people’s subjective well-being. A large body of literature has revealed that Internet use has a considerable effect—either positive or negative—on subjective feelings such as loneliness. However, the outcomes associated with Internet use in the older population still remain largely unexplored.

Prior studies have displayed contradictory findings on the relationship between Internet use and well-being. Early studies demonstrated that increased Internet use was linked to a decrease in the size of a users’ social network and an increase in depression and loneliness (Kraut et al., 1998). The *displacement hypothesis* was further proposed to explain that Internet use displaced more beneficial face-to-face socialization, thereby harming people’s relationships and well-being (Nie, 2001). The association between problematic Internet use (e.g., addiction or compulsive Internet use) and loneliness has provided additional evidence for this hypothesis (Matsuba, 2006; Ceyhan and Ceyhan, 2008; Casale and Fioravanti, 2011).

However, another line of research has revealed the positive effect of Internet use on mental health. The Internet can be useful in reducing mental health problems, including loneliness, by improving existing relationships and providing opportunities to form new ones (Valkenburg and Peter, 2007; Li and Yang, 2021). In particular, when the Internet is used for social communication, it is correlated to lower levels of loneliness among the older population (Sum et al., 2008; Erikson and Johnson, 2011). Studies have shown that social network sites, such as Facebook, can help older adults not only to strengthen family ties but also to maintain and expand their non-family ties (Yu et al., 2016). The positive effect of Internet use is consistent with the *stimulation hypothesis*, which emphasizes the important role of interactive online activity (Nowland et al., 2018). In contrast to passively obtaining information from the Internet, online interactions or communications can help people build social connections and facilitate higher quality of their face-to-face relationships, which consequently improves their well-being.

We conjecture that the seemingly contradictory findings on the consequences of Internet use can be attributed to different types of Internet use. It is believed that whether Internet behaviors are good for well-being hinges on whether the behaviors advance or thwart innate human desires (Clark et al., 2018). Connection-promoting Internet users who use Internet to communicate with others may benefit from increased perceived connectedness and social support, which promotes their well-being (Ahn and Shin, 2013; Liu and Yu, 2013). Non-connection-promoting Internet use, such as obtaining information and entertainment, may satisfy some human desires, such as the need for knowledge and relaxation (Gardner et al., 2005). However, non-connection-promoting Internet use fails to contribute to interpersonal connections and does not fulfill needs for acceptance and belonging, which has negative effects on well-being (Antunes et al., 2022).

Previous studies also suggest that it is necessary to understand differentiated Internet use among older adults (Schehl et al., 2019) and problematic smartphone usage, such as extensive usage, which is negatively correlated with subjective well-being (Horwood and Anglim, 2019). Thus, it is vitally important to distinguish the different effects of specific online behaviors. Since Internet use behaviors vary between cultures and age groups (Barbosa Neves et al., 2018; Hunsaker and Hargittai, 2018), our study investigates how different online behaviors relate to the loneliness experienced by the older population in China. Specifically, we classified online behaviors into two dimensions: interpersonal communication and information acquisition. Interpersonal communication, namely using the Internet to communicate with others, is a process of “person–machine–person” interaction for realizing social Internet use, which is a form of connection-promoting use (Ihm and Hsieh, 2015). Information acquisition, namely using the Internet to access information, is a process of “person–machine” interaction for realizing instrumental Internet use, which is a form of non-connection-promoting use (Hunsaker and Hargittai, 2018).

Although previous research has provided qualitative explanations for the positive and negative effects of Internet use, limited empirical work has been conducted to explore the underlying mechanisms, especially for older adults. A potential underlying mechanism is the social network. Social networks are often used to evaluate an individual’s social support and their social sources embedded in their interactions within the network (Lin, 2008). The social engagements of older adults are often reduced due to the loss of social roles, contacts, and physical abilities. Therefore, maintaining social connections and developing new ones play an important role in advancing the social well-being of older adults (Yu et al., 2016).

The social convoy model has identified and stressed two important types of social networks among older adults—family ties and friendship ties (Chopik, 2017)—on the basis of the closeness of the relationships within those networks. The relationships with people in the innermost circle of the convoy, such as spouses and core family, should be more stable than those with members of the outer circle. This characteristic is familial and natural (Antonucci et al., 2014), and will not change markedly due to individuals’ behaviors. However, friendship ties, an outer circle of social networks formed by acquaintances, coworkers, and neighbors, are believed to be less stable (Antonucci and Akiyama, 1987). Such relationships are optional and need to be maintained by frequent contacts. The strength of friendship ties will vary according to the efforts made in communication (Guiaux et al., 2007). Internet use behaviors might have different impacts on family vs. friendship ties.

Studies in the context of Western countries have consistently shown that friendship interactions are positively related to self-esteem, morale, and mental health among older adults, while family ties are not always beneficial; their effects are dependent on the quality of the relationship (Lee and Shehan, 1989; Chopik, 2017). However, previous research has also shown that family ties are more important for older adults’ well-being in non-Western cultures (Alegria et al., 2007; Lee et al., 2018). It is still unknown whether Internet use can help to enhance older adults’ social networks, including family and non-family ties, and further reduce their loneliness. Moreover, there has been little research on how family and friendship ties play different roles in the association between Internet use and loneliness in later life in non-Western contexts. China, a typical Asian country, places very strong emphasis on family ties and children’s filial responsibilities (Lin and Pei, 2016). Thus, this country provides an interesting context in which to study the roles of family ties and friendship ties in the relationship between Internet use and mental health among older adults.

Based on previous research, we proposed the following hypotheses:

**H1:** Both Internet use for interpersonal communication and information acquisition are associated with lower loneliness.

**H2:** Internet use for communication is associated with better social networks, including family ties and friendship ties.

**H3:** Internet use for information acquisition is associated with poor social networks, including family ties and friendship ties.

**H4:** Family ties and/or friendship ties play mediating roles in the relationship between Internet use and loneliness.

Our study contributes to the existing literature by empirically investigating how the mechanisms underlying social networks link different types of Internet use to older adults’ feelings of loneliness. Further, our study illuminates the influence of different types of Internet use on older adults’ loneliness and how these associations occur through social networks. Our findings may help inform policies to enhance the well-being of older populations in the digital era.

## Materials and Methods

### Sample

The China Longitudinal Ageing Social Survey (CLASS) is a nationally representative and prospective panel study that has surveyed more than 11,000 Chinese people aged 60 and above every 2 years since 2014 (data and documentation available at http://class.ruc.edu.cn) (Tang et al., 2020). We used the data of the 2018 survey which covered 28 provinces, autonomous regions, and municipalities in mainland China, and collected information from 11,418 older adults aged 60 and over living in 195 villages and 267 neighborhood communities.

Given that the main purpose of this investigation was to examine the effect of specific Internet use on the well-being of older adults, only the respondents who used the Internet (*N* = 2086) were included. We also excluded respondents with missing values on analytical variables (*N* = 223, 10.6%), resulting in the inclusion of 1863 older adults, comprising 899 women and 964 men.

### Measures

#### Dependent Variable

The dependent variable in this study was loneliness, which was assessed using the Three-Item Loneliness Scale which was previously used in the Health and Retirements Studies in the United States (Hughes et al., 2004). The respondents’ loneliness was evaluated by asking them “How often do you feel that you lack companionship?”, “How often do you feel left out?”, and “How often do you feel isolated from others?” We coded the frequency with which a respondent had experienced each feeling in the past week as 1 (hardly ever), 2 (some of the time), or 3 (often). The three items were summed, which produced a loneliness score ranging from 3 to 9 with a higher score indicating greater loneliness. The Cronbach’s α of the scale was 0.770.

#### Independent Variables

The first independent variable in this study was specific types of Internet use. According to users’ perceived usefulness of certain types of Internet use, Internet use was divided into two categories: interpersonal communication and information acquisition. Interpersonal communication is using the Internet to communicate with others and includes chatting *via* voice, video, and text. Information acquisition is using the Internet to access various forms of information, including reading the news, watching videos/movies, shopping, health management, making and managing investments, arranging and booking transportation, learning, and playing games. Internet use for interpersonal communication was coded as 1 if the respondent used any function of interpersonal communication, and the same applied to information acquisition. A total of 1639 (88.0%) older adults used the Internet for interpersonal communication and 1530 (82.1%) used it for information acquisition.

The second independent variable assessed social networks. We used the Lubben Social Networks Scale (LSNS) to measure the social networks used by our sample (Lubben et al., 2006). The LSNS is constructed from a set of three questions that evaluate family ties and a comparable three questions that evaluate friendship ties. The questions comprised: “How many relatives/friends do you see or hear from at least once a month?”, “How many relatives/friends do you feel at ease with to talk about private matters?”, and “How many relatives/friends do you feel close to such that you could call on them for help?”. We coded the answers given by the respondents for each question as 0 (none), 1 (one), 2 (two), 3 (three or four), 4 (five through eight), or 5 (nine or more). The three items were summed into a scale ranging from 0 to 15 for family and friendship ties. The Cronbach’s α coefficients were 0.808 and 0.887 for the subscales of family and friendship ties, respectively.

#### Controlled Variables

We controlled for sociodemographic variables in the analysis, including gender, age, marital status, living arrangements (living with children or not), education (less than secondary/secondary and above), self-rated health status (healthy/fair or unhealthy), and living area (rural or urban).

### Analytical Strategy

We conducted the analyses in three steps. In the first step we reported descriptive statistics of older adults’ loneliness, social networks, and other variables included in the multivariate models by different Internet use behavior. In the second step, we used multivariate linear regression models to examine the associations between Internet use, social networks, and loneliness. In the third step, we used bootstrap to test the mediating effects of family and friendship ties in the association between specific types of Internet use and loneliness.

## Results

### Descriptive Analysis

In Table 1, the sample’s characteristics and the differences between the older adults who used the Internet and those who did not are presented. Consistent with previous studies, older adults who use the Internet are younger, more educated, healthier, and more likely be married and live in urban areas than those who do not. However, the percentage of older adults who use the Internet and live with children is lower than those in the opposite group. Older adults using the Internet have higher mean values of family and friendship ties than those who do not, while their loneliness score is lower.

**TABLE 1:** Descriptive statistics of the sample.

	Using Internet	Not using Internet	χ^2^/*t*
Age (60–95)	66.92 (5.27)	72.32 (7.34)	30.025***
Male (%)	51.7	49.7	2.616
Married (%)	83.1	66.4	200.004***
Living with children (%)	25.6	36.2	76.501***
Secondary education or above (%)	68.3	25.8	1225.154***
Self-rated healthy (%)	55.7	42.4	108.810***
Rural (%)	26.8	63.2	817.910***
Family ties (0–15)	7.71 (2.71)	7.19 (2.85)	7.011***
Friendship ties (0–15)	7.01 (3.09)	6.18 (3.23)	10.051***
Loneliness (3–9)	4.11 (1.42)	4.60 (1.62)	−12.934***
*N*	1863	8088	

Values for categorical variables are in percentage. The mean values, followed by standard deviation in parentheses, are presented for the continuous variables. The symbol “***” indicates the statistical significance (p < 0.001) between two groups based on Chi-square test or t-test.

### Multivariate Regression Analysis

Results from the regression analyses of social networks are shown in Table 2. In models 1a and 1b, having been controlled for other covariates, Internet use for communication was positively associated with the scores for family ties (β = 0.087, *p* < 0.001) and friendship ties (β = 0.111, *p* < 0.001). The coefficient of communication in the friendship ties model is significantly larger than that in the family ties model (χ^2^ = 7.98, *p* < 0.01). In models 2a and 2b, after controlling for other covariates, Internet use for information acquisition was negatively associated with the scores for family ties (β = −0.073, *p* < 0.001) and friendship ties (β = −0.108, *p* < 0.001), but there was no significant difference between the coefficients in the friendship and family ties models.

**TABLE 2:** Social networks regressed on Internet use (β).

	Model 1a	Model 1b	Model 2a	Model 2b
	Family ties	Friendship ties	Family ties	Friendship ties
Interpersonal communication	0.087***	0.111***		
Information acquisition			−0.073**	−0.108***
Age	0.062*	0.016	0.048*	–0.003
Male	–0.003	–0.004	0.001	–0.009
Married	0.095***	0.051*	0.094***	0.05*
Living with children	0.048*	–0.001	0.046	–0.003
Secondary education or above	0.003	–0.003	0.011	0.007
Self-rated healthy	0.042	0.071**	0.041	0.07**
Rural	−0.092***	−0.143***	−0.111***	−0.159***
*R* ^2^	0.032	0.047	0.030	0.047
*N*	1863	1863	1863	1863

***p < 0.001, **p < 0.01, *p < 0.05.

The results of the regression analyses of loneliness are shown in Table 3. In model 3a, Internet use for communication was significantly associated with lower loneliness (β = −0.054, *p* < 0.05). In model 3b, the score for family ties was also significantly associated with lower loneliness (β = −0.049, *p* < 0.001), but the coefficient of friendship ties was not significant. After adding social networks into the model, the coefficient of communication declined slightly (−0.054 vs. −0.049). Models 4a and 4b address the association between Internet use for information acquisition and loneliness. In model 4a, information acquisition was negatively associated with loneliness (β = −0.057, *p* < 0.05). After adding social networks into model 4b, the coefficient of information acquisition increased (−0.057 vs. −0.062), indicating that information acquisition is likely to be negatively associated with social networks.

**TABLE 3:** Loneliness regressed on Internet use (β).

	Model 3a	Model 3b	Model 4a	Model 4b
Interpersonal communication	−0.054*	−0.049*		
Information acquisition			−0.057*	−0.062**
Age	0.076**	0.081**	0.078**	0.082**
Male	0.022	0.022	0.014	0.014
Married	−0.087***	−0.080**	−0.086***	−0.078**
Living with children	0.024	0.028	0.03	0.035
Secondary education or above	−0.060*	−0.060*	−0.066**	−0.065*
Self-rated healthy	−0.170***	−0.168***	–0.164	–0.162
Rural	−0.072**	−0.077**	−0.059*	−0.067**
Family ties (0–15)		−0.093**		−0.094**
Friendship ties (0–15)		0.028		0.017
*R* ^2^	0.067	0.072	0.067	0.074
*N*	1863	1863	1863	1863

***p < 0.001, **p < 0.01, *p < 0.05.

### Robustness Tests

To check the robustness of the results, we performed two additional analyses. We first measured the information acquisition as a continuous score to ensure the validity of our measurement. Information acquisition consisted of nine items. We coded the use of information acquisition as a continuous score (0–9) and performed the same sets of regression models. The results are shown in Table 4. They are consistent with our previous analysis that used binary measurement of information acquisition.

**TABLE 4:** Social networks and loneliness regressed on information acquisition (β).

	Model 5	Model 6	Model 7a	Model 7b
	Family ties	Friendship ties	Loneliness	Loneliness
Information acquisition (0–9)	−0.08***	−0.078***	−0.115***	−0.122***
Age	0.04	–0.008	0.063**	0.067**
Male	0.001	0.007	–0.007	–0.007
Married	0.093***	0.049**	−0.088***	−0.080***
Living with children	0.045	–0.006	0.032	0.036
Secondary education or above	0.014	0.010	−0.062*	−0.061*
Self-rated healthy	0.042	0.070**	−0.160***	−0.157***
Rural	−0.113***	−0.160***	−0.061*	−0.069**
Family ties (0–15)				−0.100***
Friendship ties (0–15)				0.019
*R* ^2^	0.031	0.042	0.077	0.087
*N*	1863	1863	1863	1863

***p < 0.001, **p < 0.01, *p < 0.05.

Considering that 88.0% of the participants used the Internet for interpersonal communication and 82.1% of the participants used the Internet for information acquisition in our sample, PSM was conducted to adjust the biased sample distribution. In the first step, all control variables were included in the logistic regression to predict the two types of Internet use. Then, radius matching was used to obtain the average treatment effects (ATT). The results of ATT and *t* values of the PSM are shown in Table 5. The PSM results are consistent with those from ordinary least squares, indicating that estimates were robust. Thus, we conclude that the biased sample distribution did not introduce estimation biases.

**TABLE 5:** Propensity score matching of two types of Internet use.

	Family ties		Friendship ties		Loneliness	
	ATT	*t*	ATT	*t*	ATT	*t*
Interpersonal communication	0.535 (0.281)	1.904	0.944 (0.358)	2.637	−0.245 (0.101)	−2.426
Information acquisition	−0.472 (0.158)	2.987	−0.813 (0.164)	−4.957	−0.260 (0.089)	−2.921

### Mediating Effects Analysis

**FIGURE 1:**
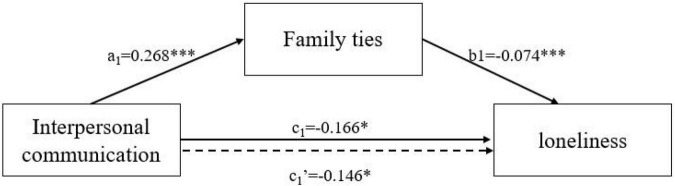
The mediating effects of family ties in the association between interpersonal communication and loneliness. ***p < 0.001, **p < 0.01, *p < 0.05.

**FIGURE 2:**
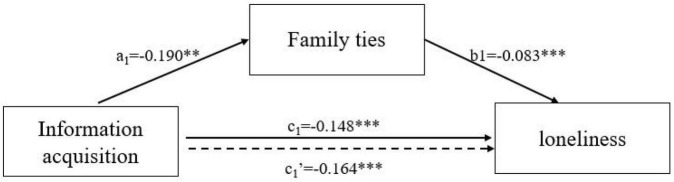
The suppressing effects of family ties in the association between information acquisition and loneliness. ***p < 0.001, **p < 0.01, *p < 0.05.

Bootstrap Model 4 of PROCESS Macro 3.5 for SPSS was used to test the potential mediating effects of family ties (since friendship ties were not significant) in the associations between different types of Internet use and loneliness (Hayes, 2017). The bias-corrected percentile bootstrap test was used to extract 5000 times to calculate the 95% confidence interval. We present the results on Table 6 and Figures 1, 2. In the mediation model of communication, a significant indirect effect of family ties (a_1_ × b_1_ = −0.020, 95% CI = [−0.039, −0.005]) was found and the direct effect of communication (c_1_′ = −0.146, *p* < 0.05) was still significant. Family ties acted as a partial mediator in the association between Internet use for communication and loneliness. In the mediation model of information acquisition, the indirect effect of family ties (a_2_ × b_2_ = 0.016, 95% CI = [0.005, 0.030]) was significant. The fact that the indirect effect of family ties was positive meant that Internet use for information acquisition was associated with higher loneliness through increasing the decline of family ties. However, the direct effect of information acquisition was significantly negative (c_2_′ = −0.164, *p* < 0.01). This shows that family ties play a suppressing role in the association between information acquisition and loneliness (MacKinnon et al., 2000; Cheung and Lau, 2008). Information acquisition can decrease feelings of loneliness in older adults, but the protective effects of information acquisition on mental health were partially offset by the decline in family ties.

**TABLE 6:** Bootstrap test of the mediating effects.

			β	LLCI	ULCI	*p*
Model 5	Interpersonal communication	Direct effect	−0.146			0.046
	Family ties	Indirect effect	−0.020	−0.039	−0.005	
Model 6	Information acquisition	Direct effect	−0.164			0.006
	Family ties	Indirect effect	0.016	0.005	0.030	

## Discussion

Our study investigated the association between specific types of Internet use and loneliness based on a national representative survey for the older population in China. We focused on how Internet use, in terms of interpersonal communication and information acquisition, affects the loneliness experienced by older adults. We further examined the mediating role of social networks, including family and friendship ties, as an underlying mechanism.

Consistent with findings from previous studies, our research suggests that Internet use for interpersonal communication is associated with lower loneliness among older adults (Sum et al., 2008). Internet use for communication partially reduces loneliness by expanding older people’s social networks and thus the results support the stimulation hypothesis (Nowland et al., 2018). Text, voice, or video online chats can remove the physical barriers that are caused by the decline in mobility that occurs in the aging process, and provide older adults with opportunities to maintain existing social networks and even form new ones (Erikson and Johnson, 2011). Social networks play a vital role in an individuals’ well-being, including the reduction of loneliness (Antonucci et al., 2014). Strong social networks, especially family ties, can effectively reduce loneliness among Chinese older adults.

The effect of Internet use for information acquisition on loneliness and social networks is different from that of interpersonal communication for Chinese older adults. Compared with people who did not use the Internet for information acquisition, older adults who did showed significantly lower loneliness scores, and had smaller social networks. Although information acquisition is negatively associated with social networks, it provides older adults with other opportunities to reduce their loneliness. The stimulation hypothesis can explain this finding in a broader way. Older adults who use the Internet for information acquisition and/or entertainment can seek the information that they need to satisfy their psychological and spiritual needs, and successful access to the Internet can improve their self-efficacy (Heo et al., 2015). All these benefits help reduce their loneliness (Fry and Debats, 2002). When older adults lack companionship, a rich spiritual life can relieve their loneliness to some extent. The use of information and entertainment to enrich their spiritual lives, instead of social networks, is beneficial to their well-being.

Conversely, Internet use for information acquisition is associated with smaller social networks, which is supported by the displacement hypothesis (Kraut et al., 1998; Nie, 2001). Older adults can seek the information and help that they need from the Internet instead of from others (Kim et al., 2017). Their online activities thus displace offline relationships among older adults and the decrease in social networks is associated with higher loneliness. According to the bootstrap tests, family ties in social networks play a suppressing role in the association between information acquisition and loneliness. As a result, the positive effect of Internet use for information acquisition on loneliness among older adults is suppressed by the decline in family ties.

Moreover, previous research has found that the associations between Internet use and psychological well-being vary by age group (Chen and Persson, 2002). Most studies have found that Internet use, either for interpersonal communication or for information acquisition, is associated with greater loneliness among younger cohorts (Erikson and Johnson, 2011). However, the effects of Internet use on loneliness are associated with usage types among older adults. For example, only Internet use for information acquisition or entertainment is associated with higher loneliness in older adults, while use for communication is associated with lower loneliness (Sum et al., 2008; Erikson and Johnson, 2011). Our research found a similar pattern in the relationship between specific types of Internet use and loneliness in older groups: older adults benefited more from Internet use than did younger cohorts (Yu et al., 2016). Due to the involuntary loss of social roles and physical abilities, the connections with the world decrease among older adults. Use of the Internet, including for interpersonal communication and information acquisition, could help such adults to maintain their connections with the world, which is empowering for older adults. Moreover, our results indicate the unique nature of Internet use behavior research in older adults. This work not only enriches understanding of the effects of Internet use, but also contributes to research with older adults.

The role of family/friendship ties in the relationship between Internet use and loneliness among older adults is noteworthy, especially in the Chinese context. The impact of Internet use on friendship ties was larger than that on family ties. Contacting family members in a more convenient way is the main reason for many older adults to start using the Internet (Yu et al., 2016). However, contact with family members through the Internet, which constitutes family ties, will not change the size of family ties due to natural kinship, whereas friendship ties are expanded significantly (Hogeboom et al., 2010). Family ties are compulsory, while friendship ties are selective and flexible and are contingent on different situations (Wrzus et al., 2013). In China, the traditional culture emphasizes filial piety so that family ties have a greater impact on psychological well-being than do friendship ties (Tang et al., 2020). In this study, we found that only family ties were associated with loneliness and acted as mediator and suppressor in the association paths of Internet use and loneliness, supporting the important role of family ties among Chinese families. Despite this, studies from other countries have emphasized the benefits of friendship (Pinquart and Sorensen, 2001; Chopik, 2017). In the future, family ties will not easily be added to one’s network due to the low fertility rate that has been ongoing for a significant time in China. The role of friendship ties in the association between Internet use and well-being in older adults will be a crucial area for future research.

Several limitations should be considered when interpreting this study’s results. First, longitudinal data are needed to clarify the causal effects of Internet use on loneliness. Second, there is a bidirectional association between Internet use and loneliness (Sum et al., 2008) and only one direction of the association was addressed in this paper. Third, more detailed and comprehensive measurements of Internet use should be made, including frequency and duration.

Despite these limitations, our study has contributed to the existing literature by examining the effects of different types of Internet use on loneliness among Chinese older adults, and further investigated the mediating role of social networks, a mechanism that has been overlooked in previous studies. Although both Internet uses for communication and information acquisition have positive effects on loneliness among older adults, communication, which helps older adults enhance their offline social networks by online person–machine–person interaction, is more important for them. Pure person–machine interaction may lead to social networks shrinking, which will have a negative impact on the well-being of older adults. Older adults should learn to use the communication function of the Internet, namely person–machine–person interaction, to enhance their offline social networks and to reduce loneliness. Importantly, government policies should remind older adults to be aware of the risks associated with Internet use for information acquisition, namely person–machine interaction, and to avoid the displacement of real communication by online activity. In short, older adults should be encouraged to learn to use the Internet reasonably and to enjoy the benefits of the digital era.

## Data Availability Statement

The original contributions presented in the study are included in the article/supplementary material, further inquiries can be directed to the corresponding author.

## Author Contributions

DT contributed significantly to framework, performed the data analyses, and wrote the part of methods and results. YJ contributed significantly to analysis and wrote the part of introduction and discussion. KZ contributed significantly to analysis and manuscript preparation. DW contributed significantly to revision. All authors contributed to the article and approved the submitted version.

## Conflict of Interest

The authors declare that the research was conducted in the absence of any commercial or financial relationships that could be construed as a potential conflict of interest.

## Publisher’s Note

All claims expressed in this article are solely those of the authors and do not necessarily represent those of their affiliated organizations, or those of the publisher, the editors and the reviewers. Any product that may be evaluated in this article, or claim that may be made by its manufacturer, is not guaranteed or endorsed by the publisher.

## Footnotes

1 https://www.statista.com/statistics/617136/digital-population-worldwide/, accessed on 23 October 2021.

2 The 48th China Statistical Report on Internet Development. China Internet Network Information Center, 2021.

3 The authors calculated the rated based on The 25th and 47th China Statistical Report on Internet Development released by China Internet Network Information Center in 2010 and 2020, and 2010 and 2020 China Statistical Year Book edited by National Bureau of Statistics of China.
